# Postnatal onset of severe growth retardation after in utero exposure to carbamazepine and phenobarbital: a case report

**DOI:** 10.4076/1752-1947-3-7300

**Published:** 2009-06-12

**Authors:** Alice Liguori, Stefano Cianfarani

**Affiliations:** 1 'Rina Balducci' Center of Pediatric Endocrinology, Department of Public Health and Cell Biology, Tor Vergata University, 00133-Rome, Italy

## Abstract

**Introduction:**

Anticonvulsant drugs taken by pregnant women to prevent seizures are among the most common causes of potential harm to the fetus. While the immediate harmful effects manifesting as congenital abnormalities are well known, the long-term effects on growth of children exposed *in utero* to antiepileptic drugs are still uncertain.

**Case presentation:**

A 7-year-old boy presented to our clinic with severe short stature. His height was 110.4 cm (−2.4 standard deviation score), with a target height of 177 cm (+0.35 standard deviation score). Height corrected for target height was −2.75 standard deviation score. He presented with mild dysmorphic facial features, hypospadias and postnatal onset of severe growth retardation. Biochemical and endocrine tests were in the normal range. The child was exposed *in utero* to both carbamazepine and phenobarbital.

**Conclusion:**

This case report shows for the first time that prenatal exposure to antiepileptic drugs may induce postnatal onset of severe growth retardation, suggesting the need for growth and endocrine monitoring of offspring exposed *in utero* to anticonvulsant drugs.

## Introduction

Epilepsy is common, affecting 0.5% to 1% of the population. Of these, a third are women in reproductive age, and approximately 1 in 250 pregnancies are exposed to antiepileptic drugs.* In utero* exposure to antiepileptic drugs can result in several different teratogenic effects including major malformations, dysmorphic facial features, intrauterine growth retardation, learning and behavioral problems. We report on a child exposed *in utero* to both carbamazepine and phenobarbital. He presented with mild dysmorphic facial features, hypospadias and postnatal onset of growth retardation.

## Case presentation

**Figure 1:**
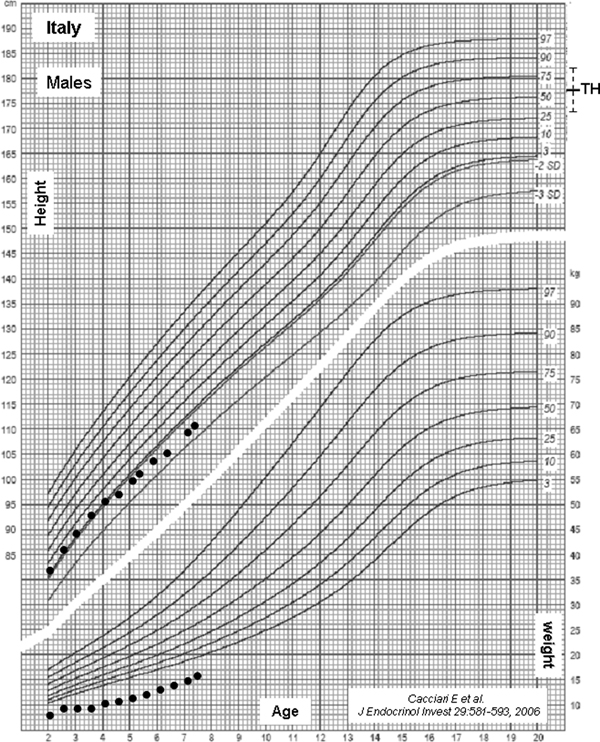
**Postnatal growth of the child exposed *in utero* to carbamazepine and phenobarbital**.

**Figure 2:**
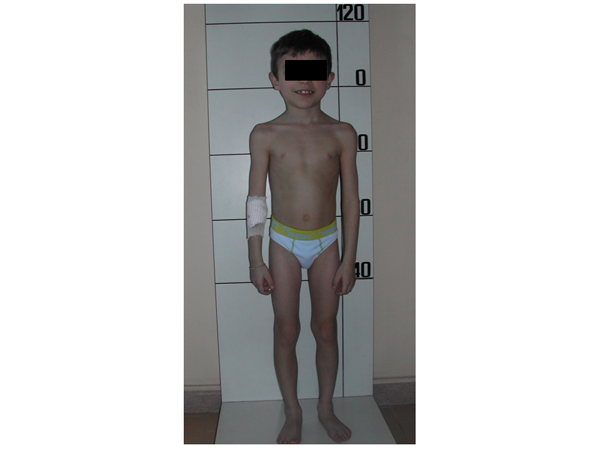
**Patient's phenotype**.

A 7-year-old boy presented to our clinic with short stature. His height was 110.4 cm (−2.4 standard deviation score (SDS)), with a target height of 177 cm (+0.35 SDS). Height corrected for target height was −2.75 SDS. His weight was 16.5 kg. Body mass index (BMI) was 13.5 (−1.8 SDS). He was born at term after an uneventful pregnancy. Birth weight was 4260 g (+1.3 SDS), birth length was 53 cm (+1.1 SDS) and birth head circumference was 37 cm (+1.3 SDS). He was born with hypospadias and underwent surgery at the age of two years. During pregnancy, his mother had undergone antiepileptic therapy with carbamazepine (200 mg bid) and phenobarbital (100 mg bid). His growth was normal during the first 12 months of age, thereafter it slowed down progressively (Figure 1). On physical examination, ocular hypertelorism, arched eyebrows, epicanthal folds, broad nasal bridge, low-set ears, and shortness of the thumb were noted (Figure 2). Bone age was six years. Neurocognitive function was normal. Liver and renal function test results, electrolytes, calcium, phosphorus, and celiac disease markers were within the normal range. Urine examination was normal and thyroid function tests were normal. Arginine and growth hormone releasing hormone (GHRH) + arginine testing showed normal growth hormone (GH) responses (GH peaks 25 εg/L and 25.3 εg/L, respectively; normal values ≥10 εg/L and 20 εg/L, respectively). Insulin-like growth factor-I (IGF-I) concentrations were in the low normal range (90 εg/L, −1.8 SDS), whereas IGFBP-3 levels were within the normal range (2.9 mg/L, +0.2 SDS). Renal and cardiac ultrasound scans were normal. Skeletal X-rays showed a short first metacarpal bone but no sign of skeletal dysplasias. Dysmorphologic evaluation did not reveal any particular syndrome. Chromosome analysis disclosed a normal 46,XY, karyotype.

## Discussion

Data on the effects of prenatal exposure to carbamazepine and/or phenobarbital are conflicting. In a retrospective study of 375 children aged from six months to 16 years born to 219 mothers with epilepsy, Kini et al. [1] reported short stature in 6.5% of children exposed to carbamazepine and 6.4% of children exposed to polytherapy. In a prospective observational study across 25 epilepsy centers in the USA and UK, Meador et al. [2] observed that more adverse outcomes were observed in pregnancies with *in utero* valproate exposure. In children exposed *in utero* to carbamazepine, the following congenital malformations have been reported: absent kidney, duplicate renal pelvis, hypospadias, and inguinal hernia. In a cohort of female patients with epilepsy, Artama et al. [3] reported that the risk for congenital malformations was not elevated in offspring of mothers using carbamazepine, oxcarbazepine, or phenytoin (as monotherapy or polytherapy without valproate). In rats, Manent et al. [4] reported that prenatal exposure to vigabatrin and valproate, which act on GABA signaling, induces hippocampal and cortical dysplasias, which are likely to result from a neuronal migration defect and neuronal death. In contrast, offspring of rats exposed to carbamazepine showed no clear-cut evidence of dysplasias. Wide et al. [5] found a significant reduction in weight, head circumference and length, which tended to improve toward the first year and was marked in babies exposed to polytherapy and also in babies exposed to carbamazepine monotherapy. However, it has to be pointed out that nearly all studies on the adverse fetal effects of antiepileptic drugs have methodological shortcomings, including retrospective or inadequately prospective design, insufficient sample size, recruitment and assessment bias, limited length of follow-up, questionable choice of controls, and failure to account for potential confounders [6].

Our case is consistent with two previous reports showing either impaired physical growth in infants exposed to anticonvulsant drugs *in utero* in spite of normal birth size [7] or increased frequency of major malformations, microcephaly, and growth retardation in infants exposed to carbamazepine compared with control infants [8]. However, the severity of growth retardation and the full investigation of GH-IGF-I axis make our case unique. The finding of reduced IGF-I levels despite normal GH peak responses to stimulation tests raises the issue of a potential disrupting effect of the *in utero* antiepileptic exposure on postnatal GH-IGF-I axis function.

## Conclusion

This case report shows for the first time that prenatal exposure to antiepileptic drugs may induce postnatal onset of severe growth retardation, thus suggesting the need for growth and endocrine monitoring of offspring exposed *in utero* to anticonvulsant drugs.

## Abbreviations

GH: growth hormone; GHRH: growth hormone releasing hormone; IGF-I: insulin-like growth factor-I; IGFBP-3: insulin-like growth factor binding protein-3.

## Consent

Written informed consent was obtained from both parents of the patient for publication of this case report and any accompanying images. A copy of the written consent is available for review by the Editor-in Chief of this journal.

## Competing interests

The authors declare that they have no competing interests.

## Authors' contributions

AL and SC followed up the patient in the clinics, performed the literature review, drafted the manuscript, and read and approved the final version of the manuscript.
